# Few-Layered MXene Modulating In Situ Growth of Carbon Nanotubes for Enhanced Microwave Absorption

**DOI:** 10.3390/molecules30071625

**Published:** 2025-04-05

**Authors:** Qing Tang, Qi Fan, Lei He, Ping Yu, Qing Huang, Yuanming Chen, Bingbing Fan, Kun Liang

**Affiliations:** 1 Nano Science and Technology Institute, University of Science and Technology of China, Suzhou 215123, China; tangqing@nimte.ac.cn; 2 Zhejiang Key Laboratory of Data-Driven High-Safety Energy Materials and Applications, Ningbo Key Laboratory of Special Energy Materials and Chemistry, Ningbo Institute of Materials Technology and Engineering, Chinese Academy of Sciences, Ningbo 315201, China; fanqi@nimte.ac.cn (Q.F.); helei@nimte.ac.cn (L.H.); huangqing@nimte.ac.cn (Q.H.); 3 School of Electronic and Information Engineering, Ningbo University of Technology, Ningbo 315211, China; yuping@nbut.edu.cn; 4 Qianwan Institute of CNITECH, Ningbo 315201, China; 5 School of Materials and Energy, University of Electronic Science and Technology of China, Chengdu 611731, China; 6 School of Materials Science and Engineering, Zhengzhou University, Zhengzhou 450001, China

**Keywords:** electromagnetic wave absorption, two-dimensional material, few-layered MXene, MWCNTs, electrophoretic deposition

## Abstract

MXene is widely used in the fields of microwave absorption and electromagnetic shielding to balance electromagnetic pollution with the development of communication technologies and human health, due to its excellent surface functional groups and tunable electronic properties. Although pure multilayered MXene has an excellent accordion-like structure, the weak dielectric loss and lack of magnetic loss result in poor microwave absorption performance. Here, we propose a strategy for the catalytic growth of CNTs by the electrophoretic deposition of adsorbed metal ions, leading to the successful preparation of Ni-MWCNTs/Ti_3_C_2_T*_x_* composites with a “layer-by-layer” structure, achieved through in situ regulated growth of CNTs. By introducing dielectric–magnetic synergy to improve the impedance matching conditions, and by regulating the diameter of the CNTs to alter the electromagnetic parameters of Ni-MWCNTs/Ti_3_C_2_T*_x_*, the 2-Ni-MWCNTs/Ti_3_C_2_T*_x_* composite achieves the best reflection loss (RL) value of −44.08 dB and an effective absorption bandwidth of 1.52 GHz at only 2.49 mm thickness. This unique layered structure and the regulation strategy provide new opportunities for the development of few-layered MXene composites.

## 1. Introduction

The rapid development of radio communication and electronic devices, as well as the widespread use of electromagnetic wave pollution, poses increasing threats to human health. Precision instruments and information security are also vulnerable to electromagnetic wave interference. Therefore, the study of advanced electromagnetic wave absorbing materials to cope with the complex electromagnetic environment has become urgent [1,2,3,4,5]. Ideal electromagnetic wave-absorbing materials should have a thin thickness, a wide absorption bandwidth, a strong absorption capacity, and be lightweight [6,7]. Currently, traditional microwave absorbing materials (MAMs), such as carbon-based materials [8], conducting polymers [9], ceramics, and magnetic metal powders [10], have struggled to fulfill all four of these requirements simultaneously. In the process of exploring new materials, researchers have discovered that the properties of two-dimensional nanomaterials, such as good electrical conductivity, a large specific surface area, rich functional groups, high mechanical strength, stability, and special structures that enable strong dielectric loss and multiple reflections [2,11,12]. These characteristics make them a promising candidate for microwave-absorbing applications.

Compared to other 2D materials, such as TMDs [13], h-BN [14,15], and graphene [16,17], MXene exhibits a higher electrical conductivity, specific surface area, and tunable electronic properties [18,19,20,21]. Its rich variety of surface functional groups and its layered structure, which are conducive to prolonged electromagnetic wave absorption, have garnered increasing attention in the field of microwave absorption (MA). In recent years, although MXene has been extensively studied, its overall MA performance is still greatly limited by its single microwave absorption mechanism, high relative complex dielectric constant, and low relative complex permeability constant. To further enhance its MA characteristics and adaptability, MXene-based composites have been developed by adjusting the composition and structural design to obtain MAMs with exceptional properties. For example, heterogeneous interfacial structures [22], dielectric–magnetic synergies [23], porous structures [24], and hyperstructures [25] have all been explored. Carbon-based materials primarily include 1D carbon nanotubes, 2D graphene [26], and 3D carbon nanospheres, which are common high dielectric loss materials. These materials have found numerous applications in the preparation of heterogeneous interfaces and high conductive loss MXene/carbon matrix composites [27,28]. The composites of one-dimensional (1D) carbon nanotubes make them more prominent in generating conductive networks and conduction loss, contributing to a higher conductive loss and polarization loss at lower amounts [17]. Magnetic transition metal atoms (such as Co, Fe, and Ni) are commonly used as catalysts to catalyze the in situ synthesis of CNTs [29,30], ensuring catalytic growth at lower temperatures while preventing oxidation of the substrate material and structural collapse caused by phase transitions. Che et al. [31] investigated the fabrication of a magnetic CNTs/Ni heterostructure decorated on an MXene substrate via a facile in situ induced growth method. Impedance matching conditions are improved by increasing interface polarization and magnetic loss through dielectric–magnetic synergy and the heterogeneous interface structure, resulting in superior microwave absorption performance of −56.4 dB at only 2.4 mm thickness. The excellent wave-absorbing performance benefits from the 3D porous structure and the dielectric–magnetic synergy. Notably, most of the MXene-based wave-absorbing materials reported so far are still multilayered MXene, while fewer studies have focused on few-layered MXene. Few-layered MXene is susceptible to self-aggregation and accumulation during assembly due to Van der Waals forces and hydrogen bonding, which reduces their stability and MA performance [32]. Furthermore, few-layered MXene has better electrical conductivity than multilayered MXene [31], and lacks the accordion-like structure found in multilayer MXenes, leading to an impedance mismatch. As a result, most electromagnetic waves are reflected away from the wave-absorbing material rather than being absorbed. Regulating the balance between the high dielectric loss and the impedance mismatch to improve impedance matching remains a significant challenge.

Herein, we propose an electrophoretic deposition strategy for the adsorption of metal catalytic ions. Ni-MWCNTs/Ti_3_C_2_T*_x_* materials with excellent wave-absorbing properties were successfully prepared by modulating the in situ growth of CNTs. During the deposition of few-layered MXene, metal catalytic ions are adsorbed between the layers and on the surface. The growth of CNTs after annealing effectively prevents the self-aggregation and accumulation of MXene during the assembly process. Additionally, the introduction of metal magnetic atoms enhances the magnetic loss and creates dielectric–magnetic synergy. The interfacial polarization loss and conductivity loss of Ni-MWCNTs/Ti_3_C_2_T*_x_* composites are controlled by adjusting the tube diameter of CNTs to improve the impedance matching conditions. This work contributes fresh concepts for the structural design of MXene-based materials and 2D nanomaterials.

## 2. Results and Discussion

### 2.1. Preparation and Characterization of Ni-MWCNTs/Ti_3_C_2_T_x_ Composites

**Figure 1:**
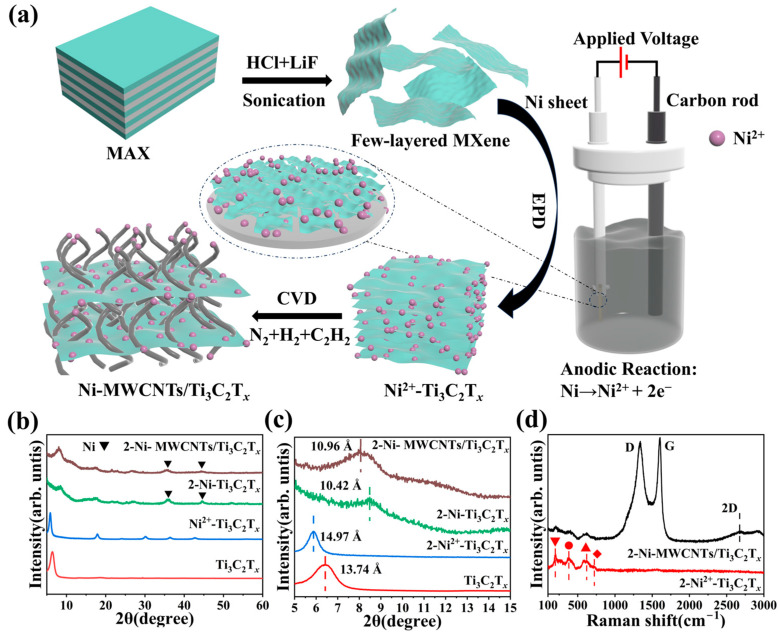
(**a**) Schematic preparation process of Ni-MWCNTs/Ti_3_C_2_T*_x_*. (**b**) XRD patterns of 2-Ni-MWCNTs/Ti_3_C_2_T*_x_*, 2-Ni-Ti_3_C_2_T*_x_*, Ni^2+^-Ti_3_C_2_T*_x_*, and Ti_3_C_2_T*_x_*. (**c**) Magnification of XRD patterns in (**b**,**d**) Raman spectra of 2-Ni-MWCNTs/Ti_3_C_2_T_x_ and Ni^2+^-Ti_3_C_2_T*_x_* collected with a 532 nm laser. ▼ A_1g_(Ti, T*_x_*), ● E_g_(T*_x_*), ▲ E_g_(C), and ◆ A_1g_(C) represent the four characteristic peaks of Ti_3_C_2_T*_x_*.

The preparation of Ni-MWCNTs/Ti_3_C_2_T*_x_* is shown in Figure 1a. Firstly, a few-layered Ti_3_C_2_T*_x_* MXene suspension was generated by in situ hydrofluoric acid etching of the MAX phase Ti_3_AlC_2_ (Figure S1a). The zeta potential was −36.65 mV (Figure S1b), which is attributed to the presence of negatively charged -F, -Cl, and -OH functional groups on the surface. The greater the absolute value of the zeta potential (greater than 30 mV), the more stable the solution [33], which favors the directional movement of the suspension under the influence of the electric field. Electrophoretic deposition (EPD) was then carried out, using the few-layered Ti_3_C_2_T*_x_* solution as the electrolyte, with graphite rods and Ni flakes serving as the cathode and anode, respectively. The Ti_3_C_2_T*_x_* nanoflakes were deposited on Ni flakes as the negatively charged surface moved toward the anode under the applied voltage. The Ti_3_C_2_T*_x_* nanoflakes were deposited onto the Ni flakes due to the negatively charged surface, which moved directionally toward the anode under the influence of the applied voltage. At the anode, the Ni flakes undergo an oxidation reaction, generating a high concentration of Ni^2+^ via dissolution around them. This Ni^2+^ is then adsorbed onto the interlayers and surfaces of the Ti_3_C_2_T*_x_* nanosheets as they are deposited towards the anode, forming a Ni^2+^-Ti_3_C_2_T*_x_* composite. Next, carbon nanotube (CNT) growth was initiated using low-pressure thermochemical vapor deposition (LPTCVD) at 550 °C with cracked acetylene as the carbon source. In the presence of Ni nanoparticles (which are fully reduced from Ni^2+^ to Ni monomers in H_2_ at 400 °C for 30 min to serve as active catalytic sites), carbon atoms near the interlayer and surface of Ti_3_C_2_T*_x_* are rearranged to catalyze the growth of CNTs. The interlayer-grown CNTs are well-connected along the Z-axis to several layers of deposited Ti_3_C_2_T*_x_*, and the CNTs are entangled and interconnected, constructing a coherent conductive network from the dispersed MXene units.

The XRD patterns of MAX and MXene are shown in Figure 1b. The Ti_3_C_2_T*_x_* (104) peaks have completely disappeared, indicating that the Al layer in the MXene was completely etched. Only the (00*l*) peak of MXene was observed, and the main peak (002) shifted to a lower angle, indicating the successful preparation of few-layered Ti_3_C_2_T*_x_* [34]. The Ni^2+^-Ti_3_C_2_T*_x_* was vacuum-dried at 60 °C, and compared to the few-layered Ti_3_C_2_T*_x_*, the (002) peak migrated to a lower angle. According to Bragg’s equation, the interlayer gap increases from 13.74 Å. to 14.97 Å. (Figure 1c), which suggests that Ni^2+^ is adsorbed in the interlayer of the few-layered Ti_3_C_2_T*_x_* nanosheets and the opened interlayer spacing facilitates the further adsorption of Ni^2+^. The XRD pattern of 2-Ni-MWCNTs/Ti_3_C_2_T*_x_* shows no peaks for Ti oxides or carbon. Compared to the 2-Ni-Ti_3_C_2_T*_x_* with no CNT growth, its XRD (002) peak shifts to a lower angle, indicating an increase in interlayer spacing, form 10.42 Å to 10.96 Å. There are also significant shifts in the (002) peaks of 1.5-Ni-MWCNTs/Ti_3_C_2_T*_x_* and 1-Ni-MWCNTs/Ti_3_C_2_T*_x_*, with the peaks increasing from 10.24 Å to 10.74 Å and 10.15 Å to 10.65 Å, respectively (Figure S2). The XRD (002) peak of Ni-MWCNTs/Ti_3_C_2_T*_x_* shifted to a lower angle as the EPD voltage increased, which may be related to the size of the Ni particles.

The Raman spectra of 2-Ni-MWCNTs/Ti_3_C_2_T*_x_* were obtained (Figure 1d). The Raman spectra of 1.5-Ni-MWCNTs/Ti_3_C_2_T*_x_* and 1-Ni-MWCNTs/Ti_3_C_2_T*_x_* are shown in Figure S3. Compared to Ni^2+^-Ti_3_C_2_T*_x_*, the G-band peak around 1580 cm^−1^, corresponding to sp^2^ hybridized carbon, and the D-band peak around 1330–1360 cm^−1^, corresponding to sp^3^ hybridized carbon atoms, were observed. The G-band indicates the degree of graphitization (i.e., the ordering and mass of the reactive CNTs), while the D-band reflects the degree of disorder in the carbon nanotubes. Additionally, a 2D peak near 2680 cm^−1^ was observed, characterizing the interlayer stacking mode or the number of layers of carbon atoms [35,36,37]. Further characterization confirmed the growth of CNTs on the surface of MXene. However, the full width at half-maximum (FWHM) of the D peak is broader and the I_D_ /I_G_ ratio is close to 1, indicating that the CNTs are less crystalline and more defective. This leads to a wider FWHM of the 2D peak and a less distinct peak shape. The Raman spectroscopic peaks of the 2-Ni^2+^-Ti_3_C_2_T*_x_* and the 2-Ni-MWCNTs/Ti_3_C_2_T*_x_* were identical to those of Ti_3_C_2_T*_x_*, with a peak at 800 cm^−1^. The four characteristic peaks of A_1g_ (Ti, T_x_), E_g_ (T_x_), E_g_ (C), and A_1g_ (C) were observed for Ti_3_C_2_T*_x_* [38]. Combined with the XRD date in Figure 1b, further analysis demonstrated that the chemical structure of the Ti_3_C_2_T*_x_* matrix remained unchanged after high-temperature annealing.

**Figure 2:**
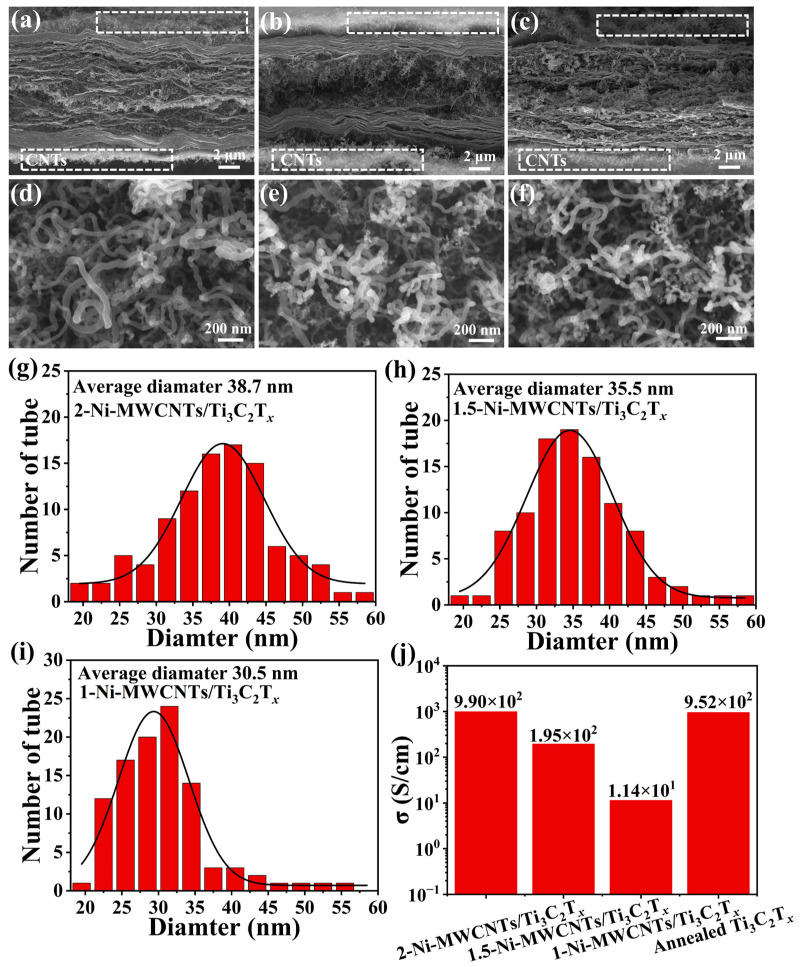
SEM images of cross section from (**a**) 2-Ni-MWCNTs/Ti_3_C_2_T*_x_*, (**b**) 1.5-Ni-MWCNTs/Ti_3_C_2_T*_x_*, and (**c**) 1-Ni-MWCNTs/Ti_3_C_2_T*_x_*. SEM images of surface from (**d**) 2-Ni-MWCNTs/Ti_3_C_2_T*_x_*, (**e**) 1.5-Ni-MWCNTs/Ti_3_C_2_T*_x_*, and (**f**) 1-Ni-MWCNTs/Ti_3_C_2_T*_x_*. MWCNTs diameter distributions grown from (**g**) 2-Ni-MWCNTs/Ti_3_C_2_T*_x_*, (**h**) 1.5-Ni-MWCNTs/Ti_3_C_2_T*_x_*, and (**i**) 1-Ni-MWCNTs/Ti_3_C_2_T*_x_* at 550 °C. (**j**) Conductivity of samples for 2-Ni-MWCNTs/Ti_3_C_2_T*_x_*, 1.5-Ni-MWCNTs/Ti_3_C_2_T*_x_*, 1-Ni-MWCNTs/Ti_3_C_2_T*_x_*, and Annealed Ti_3_C_2_T*_x_*.

The cross-sectional SEM images of 2-Ni-MWCNTs/Ti_3_C_2_T*_x_*, 1.5-Ni-MWCNTs/Ti_3_C_2_T*_x_*, and 1-Ni-MWCNTs/Ti_3_C_2_T*_x_* are shown in Figure 2a–c. As expected, with CNTs acting as the bridges and Ti_3_C_2_T*_x_* as the substrate, the in situ growth of CNTs on their interlayers and surfaces connects the Ti_3_C_2_T*_x_* in the Z-axis direction, forming an overall conductive network [31]. From the EDS (Figure S4) of the 2-Ni-MWCNTs/Ti_3_C_2_T*_x_* surface, the Ni element is relatively uniformly distributed, leading to the catalytic growth of high-density and uniformly distributed CNTs. From Figure 2d–f, it can be observed that there is a consistent pattern in the tube size distribution of the Ni-MWCNTs/Ti_3_C_2_T*_x_* samples under different deposition conditions.

In this paper, we chose to randomly measure the diameters of 100 carbon nanotubes from the surface SEM images of 2-Ni-MWCNTs/Ti_3_C_2_T*_x_*, 1.5-Ni-MWCNTs/Ti_3_C_2_T*_x_*, and 1-Ni-MWCNTs/Ti_3_C_2_T*_x_* (Figures S5–S7). The results were statistically analyzed, and the range of the carbon nanotube diameters, shown in Figure 2g–i, was found to follow a normal distribution. The average tube diameters of the CNTs for 2-Ni-MWCNTs/Ti_3_C_2_T*_x_*, 1.5-Ni-MWCNTs/Ti_3_C_2_T*_x_*, and 1-Ni-MWCNTs/Ti_3_C_2_T*_x_* were 38.7 nm, 35.5 nm, and 30.5 nm, respectively. The tube diameters of the CNTs increased with an increase in the voltage during the EPD, which was primarily influenced by the size of the Ni particles. The size of the catalyst particles typically determines the tube size of the CNTs, with larger catalyst particles leading to larger CNTs diameters [39]. During the electrophoretic deposition process, the concentration and ionic radius of the Ni^2+^ generated by Ni dissolution increased with the applied voltage [40]. As a result, the amount of Ni^2+^ adsorbed on the same-sized nickel sheet substrate also increased, and the aggregation of Ni^2+^ intensified. This led to an increase in the radius of the reduced nickel particles and the tube diameter of the grown CNTs. However, the diameter of the CNTs significantly impacts their electrical conductivity. Therefore, we characterized the electrical conductivity of 2-Ni-MWCNTs/Ti_3_C_2_T*_x_*, 1.5-Ni-MWCNTs/Ti_3_C_2_T*_x_*, and 1-Ni-MWCNTs/Ti_3_C_2_T*_x_*, and found that the conductivity increased as the CNT tube diameter increased.

**Figure 3:**
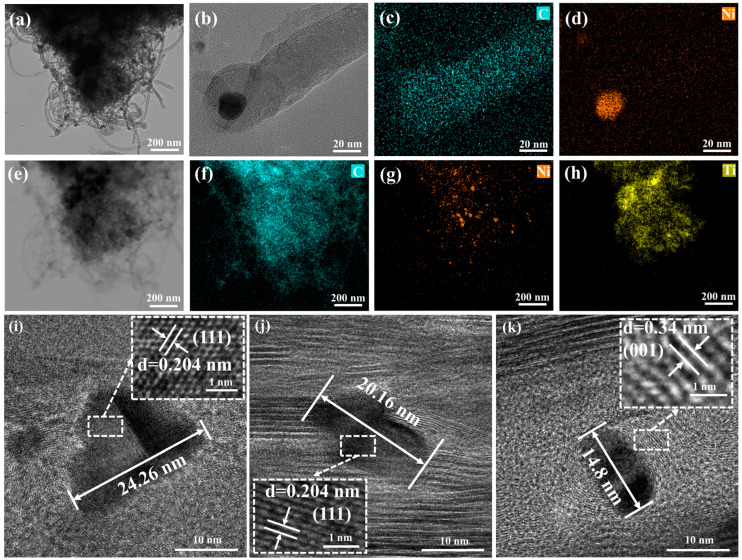
TEM images of (**a**) 2-Ni-MWCNTs/Ti_3_C_2_T*_x_*. (**b**–**d**) HRTEM images of CNTs/Ni and corresponding elemental mapping of C and Ni. (**e**–**h**) STEM images of 2-Ni-MWCNTs/Ti_3_C_2_T*_x_* and corresponding elemental images of C, Ti and Ni. HRTEM images of (**i**) 2-Ni-MWCNTs/Ti_3_C_2_T*_x_*, (**j**) 1.5-Ni-MWCNTs/Ti_3_C_2_T*_x_*, (**k**) 1-Ni-MWCNTs/Ti_3_C_2_T*_x_*.

To further demonstrate that the size of Ni particles directly affects the tube diameter of in situ-grown CNTs in Ni-MWCNTs/Ti_3_C_2_T*_x_* (Figures S8 and S9), TEM characterization was performed on the samples. The TEM image of 2-Ni-MWCNTs/Ti_3_C_2_T*_x_* is shown in Figure 3a, where CNTs are clearly seen growing epitaxially from the Ti_3_C_2_T*_x_* layers. Combined with Figure 2a and Figure S10, this confirms the in situ growth of CNTs between the Ti_3_C_2_T*_x_* layers. Additionally, Ni particles are observed at the top of the CNTs (Figure 3b). During the catalytic growth process, CNTs form as C atoms surround the Ni particles, with sp^2^ hybridized C-C bonds. Therefore, the particle size directly influences the growth behavior of CNTs. Typically, smaller Ni particles generate CNTs with smaller diameters, while larger Ni particles tend to catalyze the growth of CNTs with larger diameters. For MWCNTs, the tube diameter directly affects their electrical conductivity. Larger diameters provide more electron transport channels, leading to increased conductivity [41]. Moreover, the π-π stacking effect between the walls can enhance overall conductivity by facilitating electron coupling [42]. The interlayer Ni particles of Ni-MWCNTs/Ti_3_C_2_T*_x_* should be further characterized. The HRTEM images of 2-Ni-MWCNTs/Ti_3_C_2_T*_x_*, 1.5-Ni-MWCNTs/Ti_3_C_2_T*_x_* and 1-Ni-MWCNTs/Ti_3_C_2_T*_x_* (Figure 3i–k) clearly show Ni particle lattice fringes with a spacing of 0.204 nm, corresponding to the (111) facet of fcc Ni. The particle size of Ni increases significantly with the increase in EPD voltage, which is consistent with the CNT tube diameter distribution shown in Figure 2g–i. Furthermore, the Ni particles are clearly surrounded by a few layers of graphite lattice fringes with a thickness of 0.34 nm, corresponding to the (001) crystal plane of C. In addition, the in situ-grown MWCNTs between the Ti_3_C_2_T*_x_* layers can form a three-dimensional conductive network, enhancing electron mobility along the Z-axis and thus increasing conductivity [43]. By controlling the distribution and diameter of the MWCNTs, their electron mobility can be adjusted, thereby affecting conductivity. This is consistent with the conductivity results obtained from the experiment. As shown in Figure 2j, the conductivity of 2-Ni-MWCNTs/Ti_3_C_2_T*_x_*, 1.5-Ni-MWCNTs/Ti_3_C_2_T*_x_* and 1-Ni-MWCNTs/Ti_3_C_2_T*_x_* increases with the increasing tube diameter of the CNTs, which is consistent with our prediction. However, compared to the annealed Ti_3_C_2_T*_x_*, only 2-Ni-MWCNTs/Ti_3_C_2_T*_x_* exhibits slightly higher conductivity. The main reason for this is that the conductivity of CNTs is not only related to their tube diameter but also influenced by their crystal structure and defects. From Figure 1d and Figure S3 Raman spectra, the I_D_/I_G_ ratio of CNTs in Ni-MWCNTs/Ti_3_C_2_T*_x_* is close to 1, indicating the presence of many defects that limit free electron transport [44], which counteracts the positive effect of the increased tube diameter. The Ni 2p pattern of 2-Ni-MWCNTs/Ti_3_C_2_T*_x_* XPS is shown in Figure S11, the peaks at 853.6 and 870.7 eV belong to Ni^0^ in Ni2p_3/2_ and Ni2p_1/2_, respectively. The combination of the STEM image (Figure 3g) and the XRD pattern (Figure 1a) confirms that the Ni^2+^ has been successfully reduced to Ni particles. The C 1s spectra of 2-Ni-MWCNTs/Ti_3_C_2_T*_x_* shows three peaks at 284.8, 285.6 and 286.6 eV, with distributions corresponding to C-C, C=C and C-O bonds (Figure S12) [45].

### 2.2. Electromagnetic Parameter Analysis and Microwave Absorption Properties

**Figure 4:**
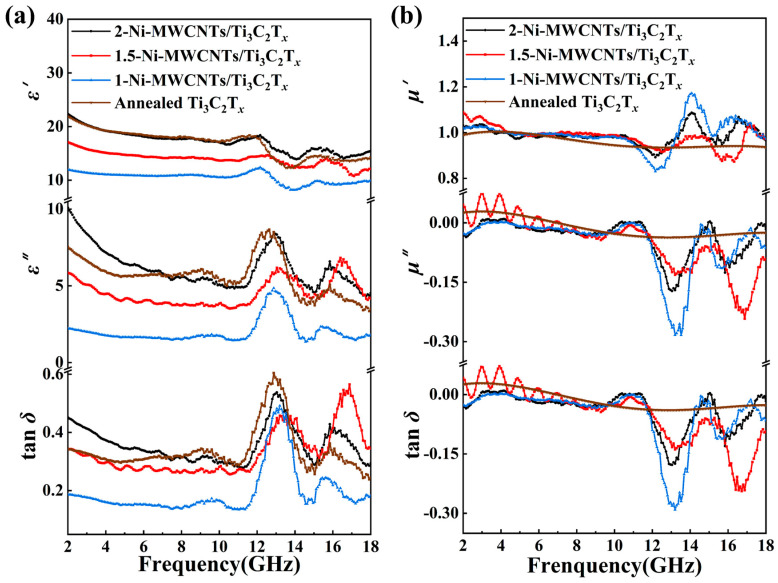
(**a**) Complex permittivity (real part (*ε*′), imaginary part (*ε*″) and dielectric loss (tan *δ* = *ε*″/*ε*′) and (**b**) Complex permeability (real part (*μ*′), imaginary part (*μ*″) and magnetic loss (tan *δ* = *μ*″/*μ*′) of 2-Ni-MWCNTs/Ti_3_C_2_T*_x_*, 1.5-Ni-MWCNTs/Ti_3_C_2_T*_x_*, 1-Ni-MWCNTs/Ti_3_C_2_T*_x_*, Annealed Ti_3_C_2_T*_x_* in paraffin matrix with a loading of 35 wt%.

To investigate the electromagnetic parameters of Ni-MWCNTs/Ti_3_C_2_T*_x_* composites, the variation of the complex dielectric constant of 2-Ni-MWCNTs/Ti_3_C_2_T*_x_* composites with different mass ratios was studied (Figure S13). The results showed that as the content of 2-Ni-MWCNTs/Ti_3_C_2_T*_x_* increased, the complex dielectric constants (*ε*′ and *ε*″) significantly increased, while the dielectric loss tangent (tan *δ*) also markedly increased. This indicates that the mass ratio has a direct effect on the dielectric constant of the composite material. However, excessively high or low dielectric constants can lead to impedance mismatch between the material and free space, which is detrimental to the effective incidence of electromagnetic waves and energy dissipation [46]. Therefore, we ultimately selected 35 wt% as the filler ratio for subsequent electromagnetic parameter testing. Subsequently, we further investigated the electromagnetic parameters of composite systems with filler ratios of 35 wt% 2-Ni-MWCNTs/Ti_3_C_2_T*_x_*, 1.5-Ni-MWCNTs/Ti_3_C_2_T*_x_*, and 1-Ni-MWCNTs/Ti_3_C_2_T*_x_* mixed with paraffin in the 2~18 GHz frequency range. To explore the relationship between the CNT diameter in the microstructure and the electromagnetic parameters, we analyzed the complex dielectric constant, complex permeability, dielectric loss tangent, and magnetic loss tangent of Ni-MWCNTs/Ti_3_C_2_T*_x_* composites with different nanotube diameters. The related data are shown in Figure 4. Specifically, the average *ε*′ values for 2-Ni-MWCNTs/Ti_3_C_2_T*_x_*, 1.5-Ni-MWCNTs/Ti_3_C_2_T*_x_*, 1-Ni-MWCNTs/Ti_3_C_2_T*_x_* and Annealed Ti_3_C_2_T*_x_* are 17.23, 14.51, 10.46, and 16.86, respectively, while the average *ε*″ values are 6.15, 5.04, 2.01, and 5.64. The real part of the dielectric constant (*ε*′) is primarily attributed to the material’s polarization effect, while the imaginary part (*ε*″) is mainly associated with the material’s electrical conductivity [47]. Dielectric performance analysis shows that the 2-Ni-MWCNTs/Ti_3_C_2_T*_x_* sample exhibits the highest real and imaginary parts of the complex dielectric constant (*ε*′ and *ε*″). This can be attributed to several synergistic effects: the wide diameter of the CNTs (38.7 nm) helps form a high-density three-dimensional conductive network, thereby improving the material’s conductivity and dielectric loss, which leads to an increase in the imaginary part of the dielectric constant. This is consistent with the trend observed in the conductivity data shown in Figure 2j. Additionally, the interface polarization (CNTs/CNTs, MXene/CNTs) and dipole polarization effects at the CNT and MXene heterojunctions further enhance the real part of the complex dielectric constant [31]. These interfacial polarization effects are closely related to the size variation of the CNTs. As the diameter of the CNTs in Ni-MWCNTs/Ti_3_C_2_T*_x_* increases, both the real and imaginary parts of the dielectric constant increase significantly, in line with the previously discussed trends. It is noteworthy that as the CNT diameter increases, the dielectric loss tangent (tan *δ*) also increases (as shown in Figure 4a), which is in line with the enhanced dielectric loss capability of the material. Furthermore, due to the introduction of magnetic Ni nano-particles, the real and imaginary parts of the permeability are not at 1 and 0, respectively, but the imaginary part of the permeability was found to be negative (Figure 4b). This may be because the Ni/MWCNTs in the Ni-MWCNTs/Ti_3_C_2_T*_x_* composites entangle with each other to form closed loops, generating eddy currents inside the composite that resist changes in the frequency of the external magnetic field in the alternating magnetic field, resulting in negative magnetic permeability [48].

**Figure 5:**
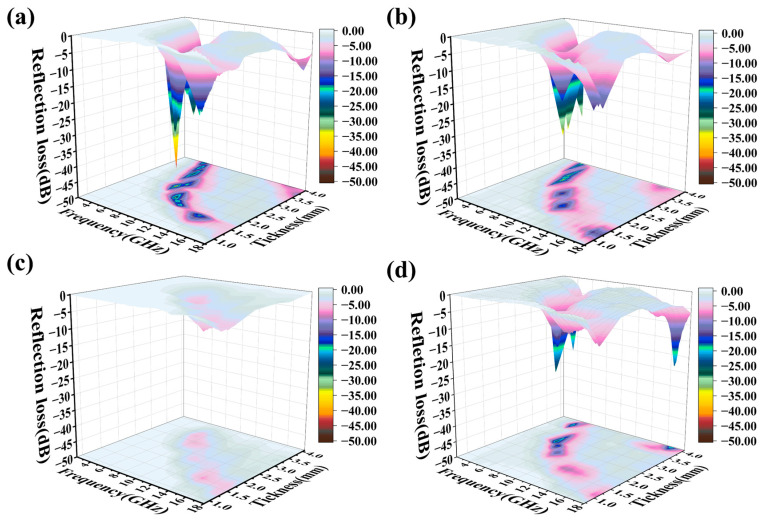
3D RL of (**a**) 2-Ni-MWCNTs/Ti_3_C_2_T*_x_*, (**b**) 1.5-Ni-MWCNTs/Ti_3_C_2_T*_x_*, (**c**) 1-Ni-MWCNTs/Ti_3_C_2_T*_x_* and (**d**) Annealed Ti_3_C_2_T*_x_* versus frequency and sample thickness.

To evaluate the electromagnetic wave absorption characteristics of the Ni-MWCNTs/Ti_3_C_2_T*_x_* composite system, the reflection loss (RL) of 2-Ni-MWCNTs/Ti_3_C_2_T*_x_*, 1.5-Ni-MWCNTs/Ti_3_C_2_T*_x_*, 1-Ni-MWCNTs/Ti_3_C_2_T*_x_*, and annealed Ti_3_C_2_T*_x_* was systematically studied. Based on the measured complex dielectric and magnetic permeability parameters, the reflection loss was calculated using transmission line theory and a metal backplane model, based on the measured dielectric and magnetic permeability parameters, with the calculation equation provided in the methodology section [49]. Figure 5 show the reflection loss of these materials at different thicknesses. Among these, the 2-Ni-MWCNTs/Ti_3_C_2_T*_x_* composite shows the best reflection loss (RL) value of −44.08 dB at 7.52 GHz and a thickness of 2.49 mm, with an effective absorption bandwidth (EAB) of 1.52 GHz (8.32~6.80 GHz). This composite exhibits the best microwave absorption performance among the Ni-MWCNTs/Ti_3_C_2_T*_x_* materials (Figure S14).

Moreover, by adjusting the thickness, the Ni-MWCNTs/Ti_3_C_2_T*_x_* composite material can meet the electromagnetic absorption requirements across the C-band (4~8 GHz) to the X-band (8~12 GHz). This significant advantage is attributed to the synergistic regulatory effect of in situ-grown CNTs within the Ti_3_C_2_T*_x_* layers and the CNTs with different diameters on the surface and the construction of a three-dimensional conductive network that regulates conductivity to optimize impedance matching. Meanwhile, the coupling effect between CNTs and magnetic Ni particles enhances multiple loss mechanisms. The CNT network effectively extends the electromagnetic wave transmission path, improving dielectric loss, while the Ni particles contribute to magnetic loss through eddy currents and hysteresis loss, achieving a dielectric–magnetic synergistic effect that improves impedance matching and optimizes electromagnetic wave absorption performance. In contrast, the best RL value of Annealed Ti_3_C_2_T*_x_* is only −26.68 dB at 6.64 GHz and a thickness of 2.71 mm. The 2-Ni-MWCNTs/Ti_3_C_2_T*_x_* and 1.5-Ni-MWCNTs/Ti_3_C_2_T*_x_* composites, compared to Annealed Ti_3_C_2_T*_x_*, benefit from the in situ-grown CNTs and Ni between the Ti_3_C_2_T*_x_* layers. This combination helps increase the polarization cross-section and extend the electromagnetic wave’s loss path, while the introduction of magnetic metal particles enhances magnetic loss [50]. The synergistic effect of dielectric and magnetic conductance improves the impedance matching, thereby enhancing the absorption performance. The absorption performance of 1-Ni-MWCNTs/Ti_3_C_2_T*_x_* is poorer, primarily due to its low dielectric loss, which results from the low dielectric imaginary part, leading to an inferior electromagnetic absorption ability. Therefore, it can be concluded that the growth and diameter of CNTs in the Ni-MWCNTs/Ti_3_C_2_T*_x_* composite play a crucial role in determining its microwave absorption performance. Subsequently, the reflection loss–frequency curves, the relationship between simulated thickness and peak frequency, and the relationship between the input impedance ratio (Z_in_/Z_0_) and electromagnetic wave frequency of Ni-MWCNTs/Ti_3_C_2_T*_x_* and Annealed Ti_3_C_2_T*_x_* were investigated, as shown in Figure S15. This study found that as the absorber thickness increased, the peak reflection loss shifted to lower frequency ranges, with the best reflection loss concentrated in the C-band (4~8 GHz). This phenomenon can be explained by the quarter-wavelength equation [51,52]:

(1) $$t_{m}=\frac{n\lambda }{4}=\frac{nc}{4f_{m}\sqrt{\left|\varepsilon_{r}\right|\left|\mu_{r}\right|}}\left(n=1,3,5,\ldots \right)$$

where *λ* is the incident wave wavelength, *c* is the speed of light in a vacuum, and |*μ_r_*| and |*ε_r_*| are the moduli of *μ_r_* and *ε_r_*, respectively. As the thickness decreases, the resonance frequency increases. When the thickness of the specimen is equal to a quarter of the incident electromagnetic wave’s wavelength, the electromagnetic waves reflected from the upper and lower surfaces of the specimen are in opposite phases. The interference causes the phases to cancel out, resulting in minimal absorption of the electromagnetic wave [49]. Moreover, the thicknesses corresponding to the minimum RL values in the experiment are mostly located near the tm curve, indicating that more electromagnetic waves are being absorbed.

### 2.3. Microwave Absorption Mechanisms

**Figure 6:**
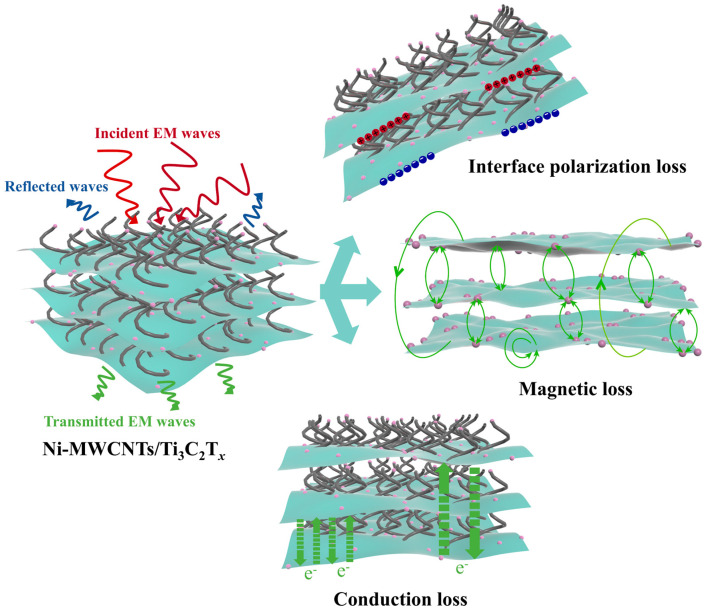
Schematic illustration of MA mechanisms for Ni-MWCNTs/Ti_3_C_2_T*_x_*.

Ti_3_C_2_T*_x_* MXene itself possesses excellent conductivity, while multiwalled carbon nanotubes (MWCNTs) form a three-dimensional conductive network through bridging interactions, further enhancing the conductivity of Ni-MWCNTs/Ti_3_C_2_T*_x_*. This continuous 2D/2D interface (Ti_3_C_2_T*_x_*/CNTs) provides an efficient channel for electron migration, significantly enhancing dielectric loss. The introduction of magnetic nano Ni particles modulates the charge distribution on the MXene surface, effectively alleviating the impedance mismatch problem caused by excessive conductivity [31,53]. At the same time, the Ni particles contribute to magnetic loss through eddy currents and hysteresis loss. The interface polarization effect between the CNT and MXene heterogeneous interfaces improves the impedance matching and optimizes the electromagnetic wave absorption performance. A schematic diagram of the electromagnetic wave dissipation mechanism of the Ni-MWCNTs/Ti_3_C_2_T*_x_* is shown in Figure 6. At a frequency of around 13 GHz (Figure 4a), the *ε*′ of 2-Ni-MWCNTs/Ti_3_C_2_T*_x_* shows a distinct valley, while *ε*″ exhibits a clear peak. Around 16 GHz, both *ε*′ and *ε*″ show peaks. This apparent fluctuation indicates the presence of substantial dielectric relaxation driven by electron motion hysteresis [54]. According to the Debye relaxation theory, the relationship between *ε′* and *ε*″ for the material is as follows [46,55]:

(2) $$\left[\varepsilon^{\prime }\left(\omega \right)-\frac{\varepsilon_{s}-\varepsilon_{\infty }}{2}\right]+{\left[\varepsilon^{\prime \prime }\left(\omega \right)\right]}^{2}={\left(\frac{\varepsilon_{s}-\varepsilon_{\infty }}{2}\right)}^{2}$$

where $\varepsilon_{s}$*,* $\varepsilon_{\infty }$ are the static dielectric constant of the material and the dielectric constant at the ultra-high limiting frequency, respectively. According to Equation (2), the imaginary part of the dielectric constant plotted against the real part (Cole–Cole curve) will form a curve with ($\frac{\varepsilon_{s}-\varepsilon_{\infty }}{2}$, 0) as the center of the circle and $\frac{\varepsilon_{s}-\varepsilon_{\infty }}{2}$ as the radius. The Cole–Cole plot of the Ni-MWCNTs/Ti_3_C_2_T*_x_* and Annealed Ti_3_C_2_T*_x_* are shown in Figure S16. The dielectric relaxation behavior of the Ni-MWCNTs/Ti_3_C_2_T*_x_* exhibits significant synergistic effects from various loss mechanisms. In the high-frequency region, the curve shows an atypical characteristic of multiple overlapping arcs, indicating the competition and coupling of various polarization mechanisms. These mechanisms include heterogeneous interface polarization, defect dipole polarization, and dielectric–magnetic coupling of magnetic nanoparticles. Some mechanisms likely exhibit synergistic effects [56]. The rich heterogeneous interfaces and defects (such as oxygen vacancies and lattice distortion) in the Ni-MWCNTs/Ti_3_C_2_T*_x_* are the primary sources of polarization loss [57]. The differences in electronegativity between the components lead to the accumulation of interface charges. For example, the -OH/-F groups on the surface of the Ti_3_C_2_T*_x_* form localized electric fields with the sp^2^ hybridized carbon of the MWCNTs, causing a relaxation loss under alternating electromagnetic fields [58]. Additionally, the defects generated during the catalytic growth of the carbon nanotubes by Ni nanoparticles, as well as the functional groups on the MXene surface, act as dipole centers and dissipate electromagnetic energy through orientational polarization [31,59]. In the low-frequency region, the Cole–Cole curve exhibits a shape resembling a straight line with a “tail” phenomenon, indicating that conductive loss dominates. The three-dimensional conductive network formed by the MWCNTs/Ti_3_C_2_T*_x_* directly converts electromagnetic energy into Joule heat through the migration of free charge carriers. Its unique interlayer through-structure significantly reduces surface reflectivity and extends the loss path of electromagnetic waves, achieving excellent impedance matching [43,60]. Moreover, the introduction of magnetic Ni nanoparticles enhances magnetic loss. These magnetic Ni nanoparticles are densely adsorbed and uniformly distributed within the layers and on the surface of MXene. This maximizes the effect of the magnetic nanoparticles, thereby enhancing magnetic loss and significantly improving the absorption performance of the Ni-MWCNTs/Ti_3_C_2_T*_x_*. In contrast, the Cole–Cole curve of the Annealed Ti_3_C_2_T*_x_* resembles a straight line, indicating the presence of only dielectric loss, with a single loss mechanism.

## 3. Experimental Section

### 3.1. Materials

Ti_3_AlC_2_ (400 mesh, 99%) was purchased from Laizhou Kai Kai Ceramic Materials Co., Ltd. (Laizhou, China). High-purity Ni flakes (>99.96%) were purchased from Zhengchen Metal Materials Co., Ltd. (Suzhou, China). HCl was purchased from Sinopharm Chemical Reagent Co., Ltd. (99%). Hexane (>99%), HF (49 wt%) and Paraffin (>99%) was purchased from Shanghai Aladdin Biochemical Technology Co., Ltd. (Shanghai, China). All chemicals were used as received, without further purification.

### 3.2. Preparation of Few-Layered Ti_3_C_2_T_x_ MXene Suspension

The few-layered Ti_3_C_2_T*_x_* MXene was fabricated though selective etching of the Al layer from MAX phase Ti_3_AlC_2_ powder using in situ-generated HF etchant, followed by ultrasonic exfoliation in deionized water (DI). Briefly, 2 g of LiF was stirred with 40 mL of 9 mol L^−1^ hydrochloric acid in a Polytetrafluoroethylene (PTFE) beaker at room temperature for 15 min. Then, 2 g of Ti_3_AlC_2_ powder was slowly added to the beaker and kept at 35 °C for 24 h. Subsequently, the suspension was washed several times with deionized water until the pH was greater than 6. The neutral solution was sonicated in an ice–water bath for 1 h to obtain as many monolayers or few layers of nanosheets as possible. Finally, the dark green solution was centrifuged at 5000 rpm for 30 min. The upper black colloidal solution was collected, and its concentration was calculated by vacuum filtration.

### 3.3. Preparation of Ni^2+^-Ti_3_C_2_T_x_ Membrane

The Ni^2+^-Ti_3_C_2_T*_x_* MXene membrane was prepared by electrophoretic deposition (EPD) using a 1.5 × 1.5 mm nickel sheet as the working electrode, a graphite rod as the counter electrode, and an electrolyte of 3.0 mg/mL Ti_3_C_2_T*_x_* suspension. The deposition was carried out at a constant voltage for 8 h. Subsequently, the deposited nickel sheet was put in a vacuum oven at 60 °C for 10 h. The Ni^2+^-Ti_3_C_2_T*_x_* film was obtained by detaching the deposit from the nickel sheet. In this paper, constant voltages of 1 V, 1.5 V, and 2 V were applied, and the resulting Ni^2+^-Ti_3_C_2_T*_x_* films were designated as 1-Ni^2+^-Ti_3_C_2_T*_x_*, 1.5-Ni^2+^-Ti_3_C_2_T*_x_*, and 2-Ni^2+^-Ti_3_C_2_T*_x_*, respectively. The nickel flakes were ultrasonically cleaned with 1 M hydrochloric acid, ethanol, and deionized water, and then vacuum-dried at 60 °C for 4 h.

### 3.4. Preparation of Ni-MWCNTs/Ti_3_C_2_T_x_

In situ MWCNTs were grown on surfaces and interlayers of Ti_3_C_2_T*_x_* using low-pressure thermal chemical vapor deposition (LPTCVD). Typically, the Ni^2+^-Ti_3_C_2_T*_x_* film was placed in a quartz crucible and introduced into CVD furnace. The chamber was first pumped to a pressure of less than 100 Pa at room temperature, purged with N_2_ for 15 min, and then heated while passing N_2_ and H_2_ at flow rates of 150 sccm and 50 sccm, respectively. The chamber was heated from room temperature to 400 °C at a rate of 5 °C/min and held at 400 °C for 30 min to allow for the reduction of nickel ions to nickel monomers. The temperature was then increased at a rate of 10 °C/min to 550 °C and held for 120 min. At 550 °C, the chamber was pumped with C_2_H_2_, and the pressure was maintained at 5 × 10^4^ Pa with a flow rate of 50 sccm. The flow rate of N_2_ was adjusted to 100 sccm, while the flow rate of H_2_ remained steady, resulting in a total gas flow rate of 200 sccm. After the holding period, C_2_H_2_ and H_2_ were turned off, and the chamber was cooled to room temperature in an N_2_ atmosphere with a flow rate of 200 sccm. The samples after in situ growth of carbon nanotubes on 1-Ni^2+^-Ti_3_C_2_T*_x_*, 1.5-Ni^2+^-Ti_3_C_2_T*_x_*, and 2-Ni^2+^-Ti_3_C_2_T*_x_* were designated as 1-Ni-MWCNTs/Ti_3_C_2_T*_x_*, 1.5-Ni-MWCNTs/Ti_3_C_2_T*_x_*, and 2-Ni-MWCNTs/Ti_3_C_2_T*_x_*, respectively. The preparation of Ni-Ti_3_C_2_T*_x_*, compared to Ni-MWCNTs/Ti_3_C_2_T*_x_*, involves replacing C_2_H_2_ with H_2_, while keeping the other LPTCVD process parameters unchanged. The preparation of Annealed Ti_3_C_2_T*_x_* is performed using a vacuum filtration membrane for LPTCVD, with the LPTCVD process parameters being the same as those for Ni-Ti_3_C_2_T*_x_*.

### 3.5. Characterization

Powder wide-angle XRD (XRD, ADVANCE D8, Bruker, Billerica, MA, USA) was performed on the samples using Cu Kα radiation (λ = 0.15406 nm) at 40 kV and 20 mA. The microstructure and composition were observed using a thermal field emission scanning electron microscope (SEM, 8230, Hitachi, Tokyo, Japan) equipped with an energy-dispersive spectroscopy (EDS) system. Transmission electron microscopy (TEM, Talos F200X, Thermo Fisher Scientific, Waltham, MA, USA) was used to identify samples’ morphology and elemental mapping. A Helios-G4-CX focused ion beam (FIB) was used to prepare the various foils. X-ray photoelectron spectroscopy (XPS, AXIS SUPRA+, SHIMADZU (CHINA) Co., Ltd., Kyoto, Japan) was performed in an XPS system with a monochromatic Al X-ray source, and the binding energy (BE) scales were assigned by adjusting the C 1s peak at 284.8 eV. Raman spectra were measured using a laser confocal Raman microscope with a He-Ne laser as an excitation line (K = 532 nm). Conductivity was measured using the AUTOMATIC FOUR POINT PROBE METER from FOUR DIMENSIONS, INC (Los Angeles, CA, USA). Electrophoretic deposition was performed using an electrochemical workstation (Bio-logic VMP3e, Seyssinet-Pariset, France) for constant voltage deposition.

The electromagnetic (EM) parameters (the complex permittivity $\varepsilon_{r}=\varepsilon^{\prime }-j\varepsilon^{\prime \prime }$ and the complex permittivity $\mu_{r}=\mu^{\prime }-j\mu^{\prime \prime }$) of all the as-prepared samples were analyzed using an E5063A vector network analyzer. The as-prepared samples were homogeneously mixed with paraffin matrix and further pressed to form coaxial rings with an outer diameter of 7 mm and an inner diameter of 3.04 mm. According to transmission theory, the reflection loss (RL) values were calculated using the following equations [31,61]:

(3) $$Ζ=\left|\frac{Z_{in}}{Z_{0}}\right|=\sqrt{\mu_{r}/\varepsilon_{r}}tanh\left[j\left(\frac{2\pi fd}{c}\right)\sqrt{\varepsilon_{r}\mu_{r}}\right]$$

(4) $$RL=20log\left|\frac{\left(Z_{in}-Z_{0}\right)}{\left(Z_{in}+Z_{0}\right)}\right|$$

where $Z_{in}$, $Z_{0}$, *c*, *d*, and f represent input impedance of the absorber, the characteristic impedance of free space, the velocity of light, the thickness of the absorbers, and incident EMW frequency, respectively.

## 4. Conclusions

In summary, Ni-MWCNTs/Ti_3_C_2_T*_x_* composites, which in situ regulate the tube diameter of CNTs and the unique layer-by-layer structure, are prepared using the catalytic strategy of electrophoretic deposition of adsorbed metal ions, resulting in excellent wave-absorbing properties. The 2-Ni-MWCNTs/Ti_3_C_2_T*_x_* composites exhibit the best reflection loss (RL) value of −44.08 dB and an effective absorption bandwidth of 1.52 GHz at a thickness of only 2.49 mm. The intertwined CNTs effectively connect the Z direction of the MXene, preventing the MXene from re-stacking while maintaining the high-density, uniformly dispersed Ni nanoparticles. This unusual structure reduces electromagnetic wave reflection and facilitates easier penetration of the material’s interior.

The interface polarization is enhanced by dielectric–magnetic synergy, and the magnetic loss is increased to rationally optimize the impedance matching. Additionally, the in situ modulation of the CNTs tube diameter enhances the dielectric loss of Ni-MWCNTs/Ti_3_C_2_T*_x_*, thereby regulating the wave-absorbing properties of the composite. This approach is expected to offer a new strategy for tuning the electromagnetic wave parameters and enhancing the wave-absorbing properties of other two-dimensional materials.

## Data Availability

Date is available at Supporting Materials.

## Supplementary Materials

The following supporting information can be downloaded at: https://www.mdpi.com/article/10.3390/molecules30071625/s1, Figure S1: (**a**) XRD patterns of Ti_3_AlC_2_, Multilayered Ti_3_C_2_T_x_, few-layered Ti_3_C_2_T*_x_*. (**b**) Zate potential of fewlayer Ti_3_C_2_T*_x_* MXene suspensions. Figure S2: (**a**) XRD patterns of 1.5-Ni-MWCNTs/Ti_3_C_2_T*_x_* and 1.5-Ni-Ti_3_C_2_T*_x_*. (**b**) XRD patterns of 1-Ni-MWCNTs/Ti_3_C_2_T*_x_* and 1-Ni-Ti_3_C_2_T*_x_*. Figure S3: (**a**) Raman spectra of 1.5-Ni-MWCNTs/Ti_3_C_2_T*_x_* and 1.5-Ni^2+^-Ti_3_C_2_T*_x_*. (**b**) Raman spectra of 1-Ni-MWCNTs/Ti_3_C_2_T*_x_* and 1-Ni^2+^-Ti_3_C_2_T*_x_*. Both have been collected with a 532 nm laser. ▼ A_1g_(Ti, T*_x_*), ● E_g_(T*_x_*), ▲ E_g_(C), and ◆ A_1g_(C) represent the four characteristic peaks of Ti_3_C_2_T*_x_*. Figure S4: SEM images of (**a**) 2-Ni-MWCNTs/Ti_3_C_2_T*_x_*, (**b**) 1.5-Ni-MWCNTs/Ti_3_C_2_T*_x_*, (**c**) 1-Ni-MWCNTs/Ti_3_C_2_T*_x_* and corresponding elemental images of Ti and Ni. Figure S5: SEM images of section 2-Ni-MWCNTs/Ti_3_C_2_T*_x_*. Figure S6: SEM images of section 1.5-Ni-MWCNTs/Ti_3_C_2_T*_x_*. Figure S7: SEM images of section 1-Ni-MWCNTs/Ti_3_C_2_T*_x_*. Figure S8: TEM images of (**a**) 1-Ni-MWCNTs/Ti_3_C_2_T*_x_*. (**b**–**d**) HRTEM images of CNTs/Ni and corresponding elemental mapping of C and Ni. (**e**–**h**) STEM images of 1-Ni-MWCNTs/Ti_3_C_2_T*_x_* and corresponding elemental images of C, Ti and Ni. Figure S9: TEM images of (**a**) 1.5-Ni-MWCNTs/Ti_3_C_2_T*_x_*. (**b**–**d**) HRTEM images of CNTs/Ni and corresponding elemental mapping of C and Ni. (**e**–**h**) STEM images of 1.5-Ni-MWCNTs/Ti_3_C_2_T*_x_* and corresponding elemental images of C, Ti and Ni. Figure S10: (**a**) The cross-sectional TEM image of 2-Ni-MWCNTs/Ti_3_C_2_T*_x_*. (**b**) TEM and (**c**) HRTEM images of 2-Ni-MWCNTs/Ti_3_C_2_T*_x_*. (**d**–**g**) STEM images of 2-Ni-MWCNTs/Ti_3_C_2_T*_x_* and corresponding elemental images of C, Ti and Ni. Figure S11: XPS spectra of Ni 2p in Ni-MWCNTs/Ti_3_C_2_T*_x_*. Figure S12: XPS spectra of C 1s in Ni-MWCNTs/Ti_3_C_2_T*_x_* Figure S13: Complex permittivity (real part (*ε*′), imaginary part (*ε*″) and dielectric loss (tan *δ* = *ε*″/*ε*′) of 2-Ni-MWCNTs/Ti_3_C_2_T*_x_*, in paraffin matrix with different loading. Figure S14: Compared RL curves of 2-Ni-MWCNTs/Ti_3_C_2_T*_x_*, 1.5-Ni-MWCNTs/Ti_3_C_2_T*_x_*, 1-Ni-MWCNTs/Ti_3_C_2_T*_x_* and Annealed Ti_3_C_2_T*_x_*. Figure S15: RL-frequency curves, relationship between simulation thickness and peak frequency, and relationship between Z_in_/Z_0_ and electromagnetic wave frequency of (**a**) 2-Ni-MWCNTs/Ti_3_C_2_T*_x_*, (**b**) 1.5-Ni-MWCNTs/Ti_3_C_2_T*_x_*, (**c**) 1-Ni-MWCNTs/Ti_3_C_2_T*_x_*, (**d**) Annealed Ti_3_C_2_T*_x_*. Figure S16: The Cole-Cole curves of (**a**) 2-Ni-MWCNTs/Ti_3_C_2_T*_x_*, (**b**) 1.5-Ni-MWCNTs/Ti_3_C_2_T*_x_*, (**c**) 1-Ni-MWCNTs/Ti_3_C_2_T*_x_*, (**d**) Annealed Ti_3_C_2_T*_x_*.
