# Supplementary material for: Few-Layered MXene Modulating In Situ Growth of Carbon Nanotubes for Enhanced Microwave Absorption
Source: Molecules. 2025 Apr 5;30(7):1625. doi: 10.3390/molecules30071625 (PMC11990155; doi:10.3390/molecules30071625)
Supplement: Supplementary file 1 [file molecules-30-01625-s001.zip › molecules-3538242-SI.pdf]

Supporting Information for

Few-layered MXene with In Situ Growing Modulating CNTs for Enhanced Microwave Absorption

Qing Tang [1, 3], Qi Fan [3], Lei He [3], Ping Yu [2], Qing Huang [3, 6], Yuanming Chen [4, *], Bingbing Fan [5, *], Kun Liang [3, 6, *]

[1] Nano Science and Technology Institute, University of Science and Technology of China, Suzhou 215123, P. R. China
[2] School of Electronic and Information Engineering, Ningbo University of Technology, Ningbo 315211, P. R. China
[3] Zhejiang Key Laboratory of Data-Driven High-Safety Energy Materials and Applications, Ningbo Key Laboratory of Special Energy Materials and Chemistry, Ningbo Institute of Materials Technology and Engineering, Chinese Academy of Sciences, Ningbo 315201, P. R. China
[4] School of Materials and Energy, University of Electronic Science and Technology of China, Chengdu 611731, P. R. China
[5] School of Materials Science and Engineering, Zhengzhou University, Zhengzhou, 450001, P. R. China
[6] Qianwan Institute of CNITECH, Ningbo 315201, P. R. China
Q.T and Q. F contribute equally.
*Corresponding author: ymchen@uestc.edu.cn (Y. Chen); fanbingbing@zzu.edu.cn (B.F.); kliang@nimte.ac.cn (K.L.)

Supplementary Figures

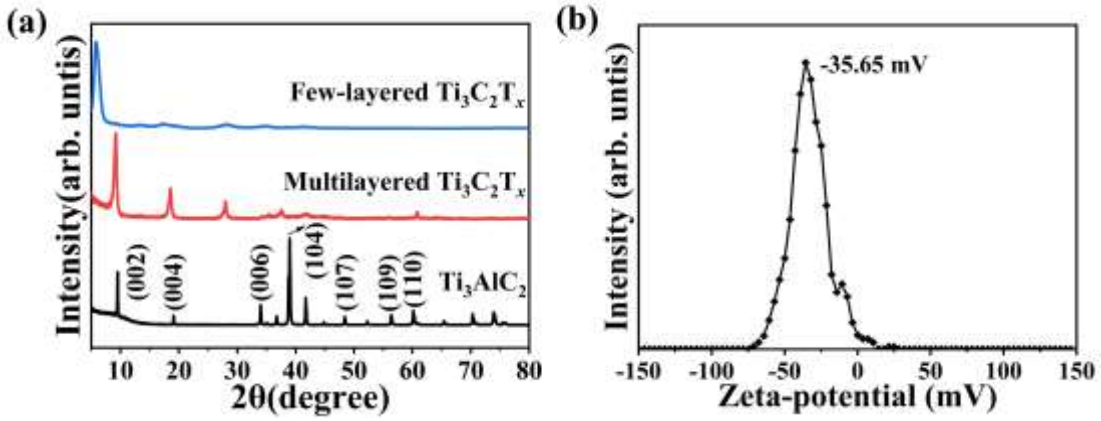

Fig. S1 **a** XRD patterns of Ti_3AlC_2, Multilayered $Ti_3C_2T_x$, few-layered $Ti_3C_2T_x$. **b** Zate potential of fewlayer $Ti_3C_2T_x$ MXene suspensions.

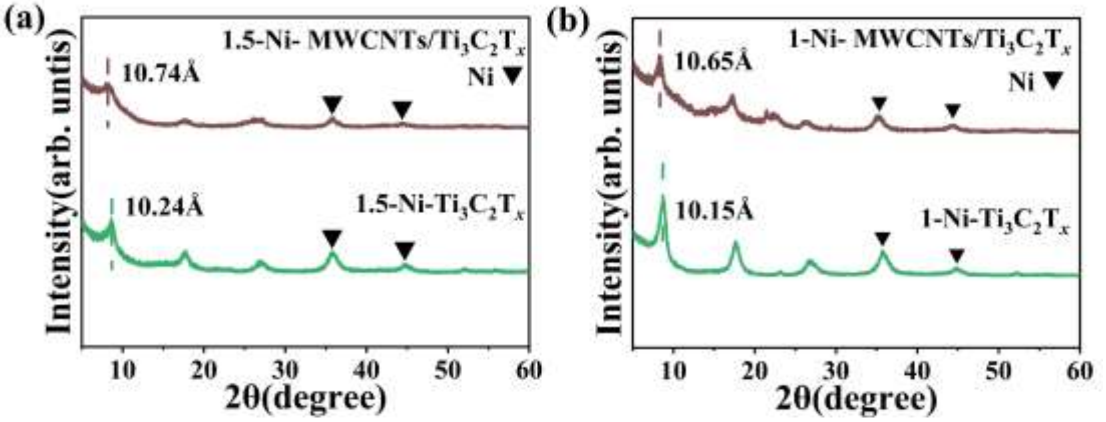

Fig. S2 **a** XRD patterns of 1.5-Ni-MWCNTs/$Ti_3C_2T_x$ and 1.5-Ni-$Ti_3C_2T_x$. **b** XRD patterns of 1-Ni-MWCNTs/$Ti_3C_2T_x$ and 1-Ni-$Ti_3C_2T_x$.

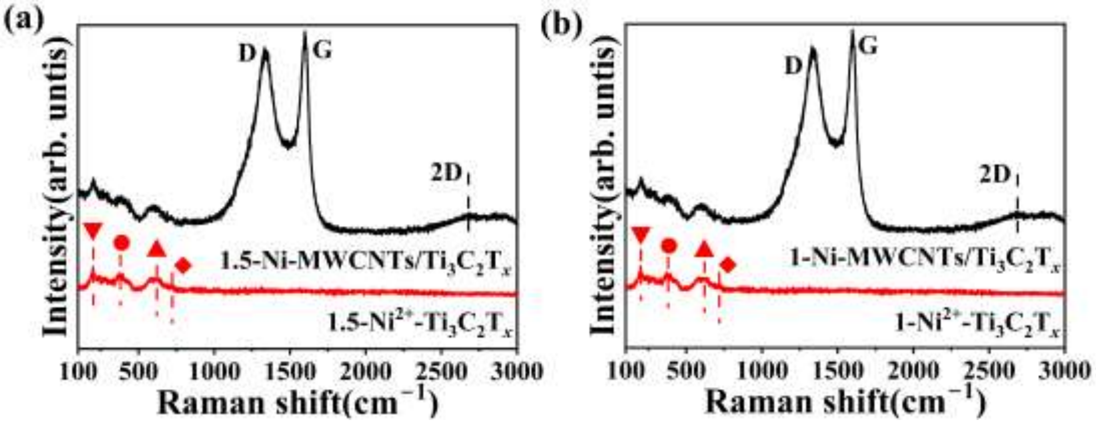

Fig. S3 **a** Raman spectra of 1.5-Ni-MWCNTs/$Ti_3C_2T_x$ and1.5-Ni^{2+}-$Ti_3C_2T_x$. b Raman spectra of 1-Ni-MWCNTs/$Ti_3C_2T_x$ and1-Ni^{2+}-$Ti_3C_2T_x$. Both have been collected with a 532 nm laser. ▼A_{1g}(Ti, T_x), ●E_g(T_x), ▲E_g(C) and ◆A_{1g}(C) represent the four characteristic peaks of $Ti_3C_2T_x$.

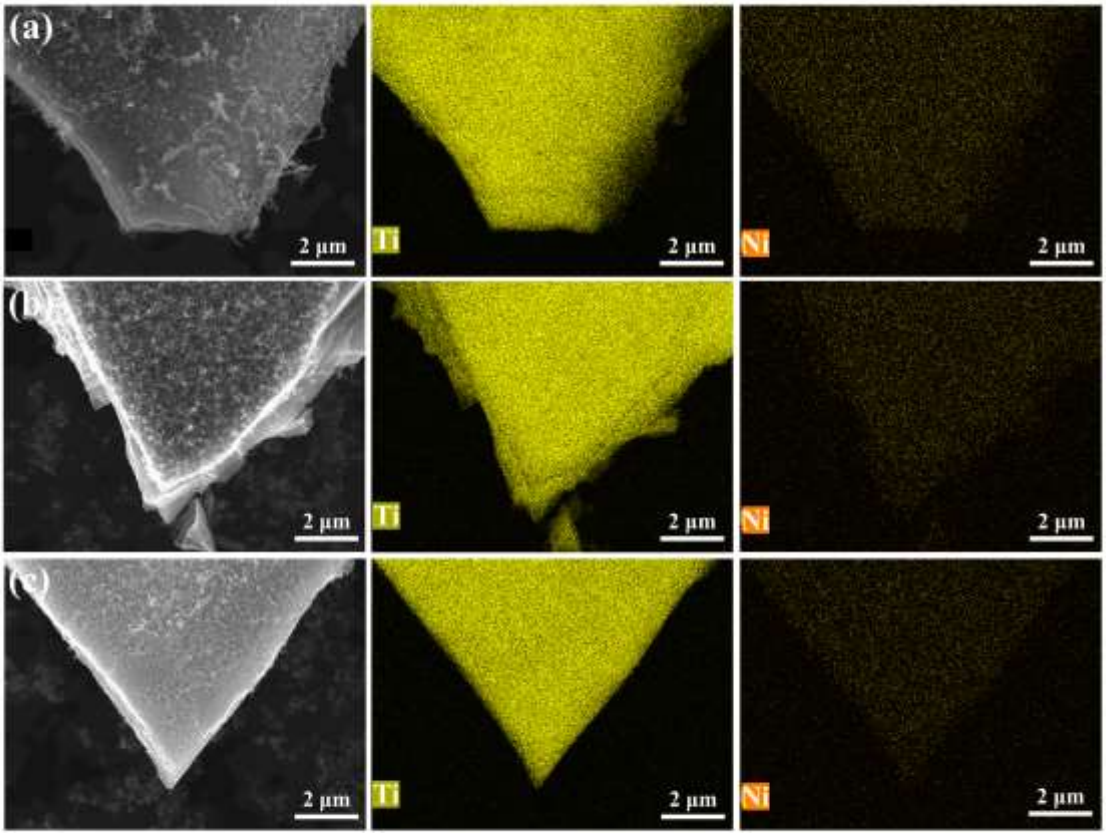

Fig. S4 SEM images of **a** 2-Ni-MWCNTs/$Ti_3C_2T_x$, **b** 1.5-Ni-MWCNTs/$Ti_3C_2T_x$, **c** 1-Ni-MWCNTs/$Ti_3C_2T_x$ and corresponding elemental images of Ti and Ni.

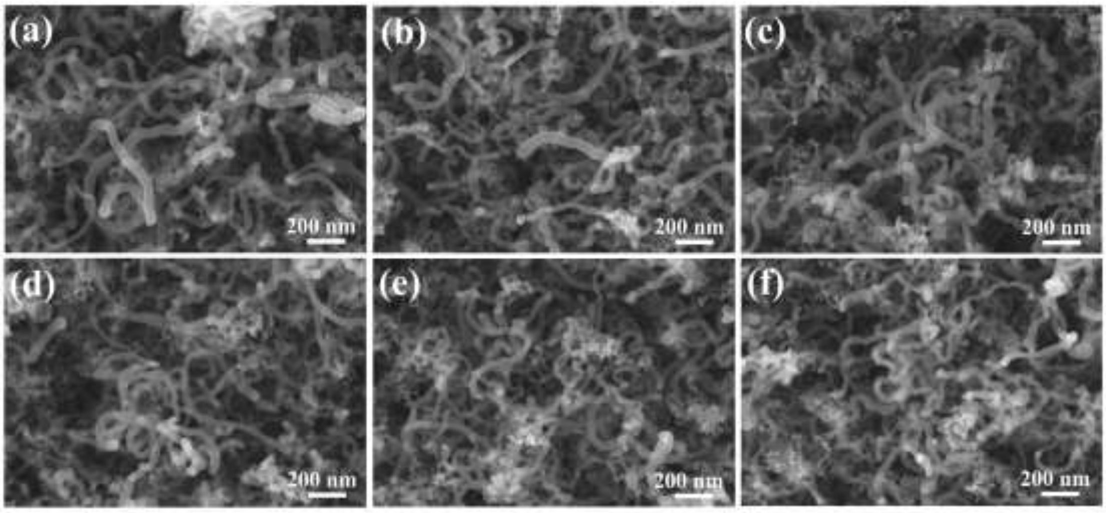

Fig. S5 SEM images of section 2-Ni-MWCNTs/$Ti_3C_2T_x$.

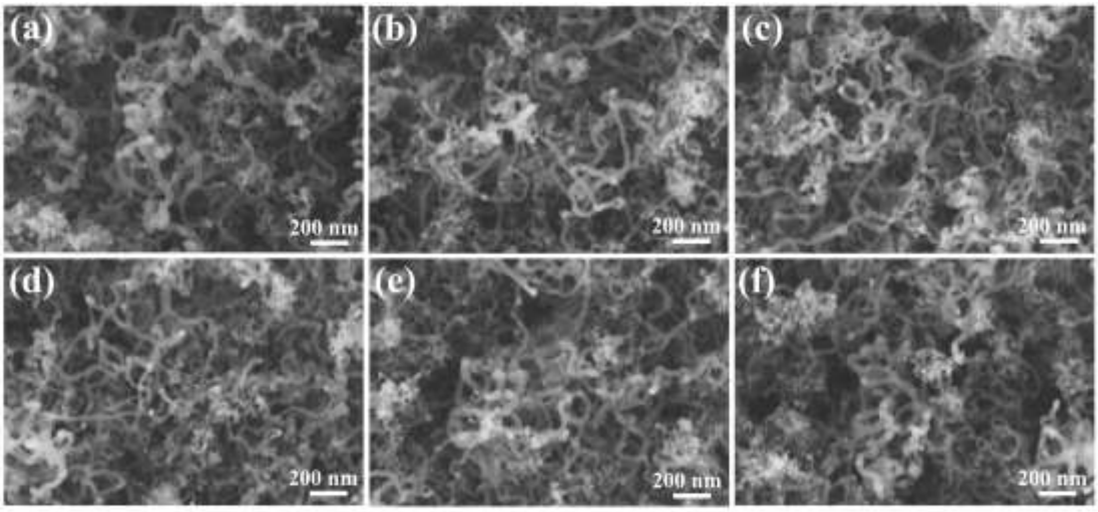

Fig. S6 SEM images of section 1.5-Ni-MWCNTs/$Ti_3C_2T_x$.

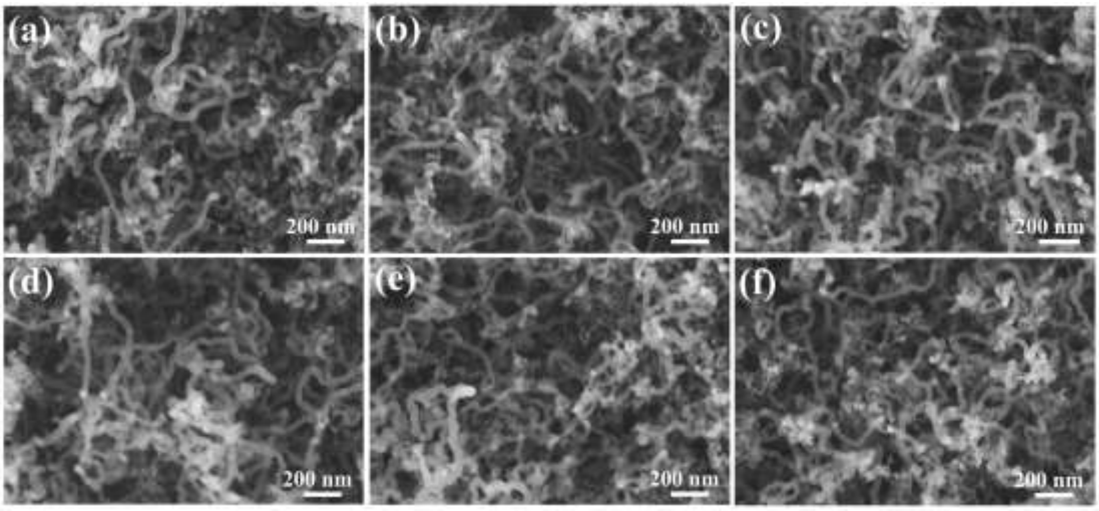

Fig. S7 SEM images of section 1-Ni-MWCNTs/$Ti_3C_2T_x$.

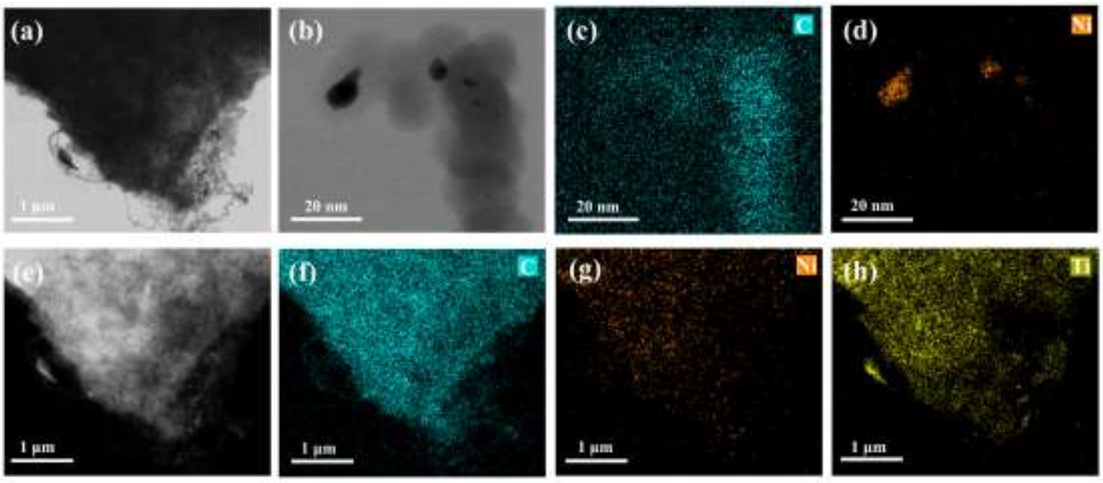

Fig. S8 TEM images of **a** 1-Ni-MWCNTs/$Ti_3C_2T_x$. **b-d** HRTEM images of CNTs/Ni and corresponding elemental mapping of C and Ni. **e-h** STEM images of 1-Ni-MWCNTs/$Ti_3C_2T_x$ and corresponding elemental images of C, Ti and Ni.

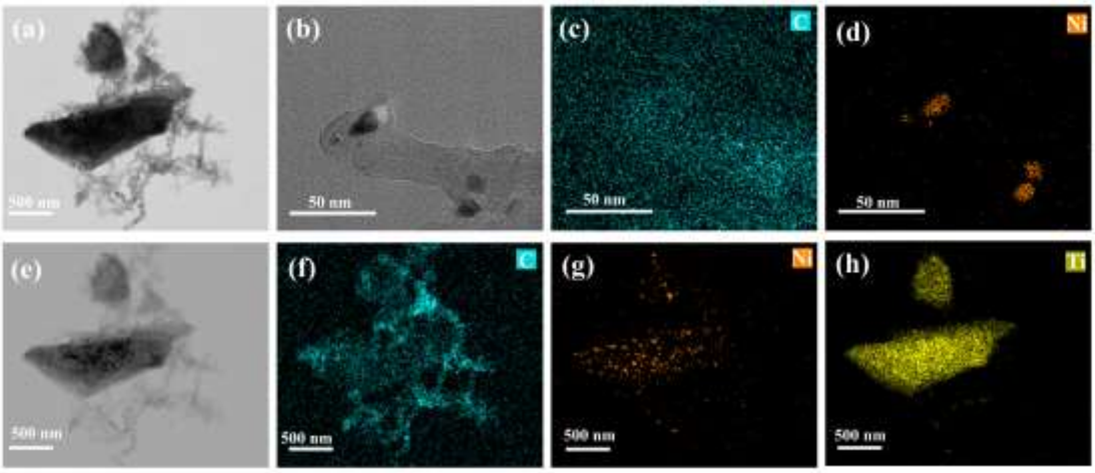

Fig. S9 TEM images of **a** 1.5-Ni-MWCNTs/$Ti_3C_2T_x$. **b-d** HRTEM images of CNTs/Ni and corresponding elemental mapping of C and Ni. **e-h** STEM images of 1.5-Ni-MWCNTs/$Ti_3C_2T_x$ and corresponding elemental images of C, Ti and Ni.

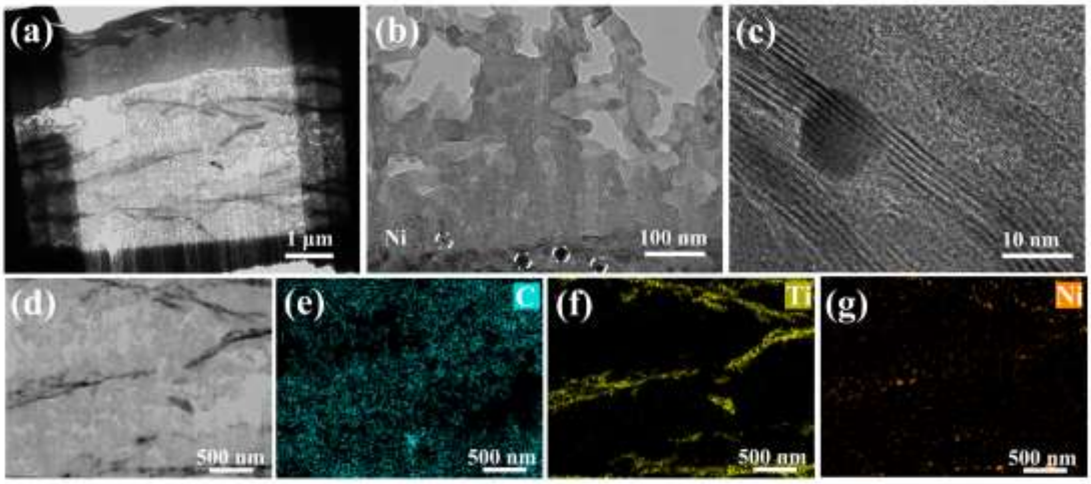

Fig. S10 **a** The cross-sectional TEM image of 2-Ni-MWCNTs/$Ti_3C_2T_x$. **b** TEM and **c** HRTEM images of 2-Ni-MWCNTs/$Ti_3C_2T_x$. **d-g** STEM images of 2-Ni-MWCNTs/$Ti_3C_2T_x$ and corresponding elemental images of C, Ti and Ni.

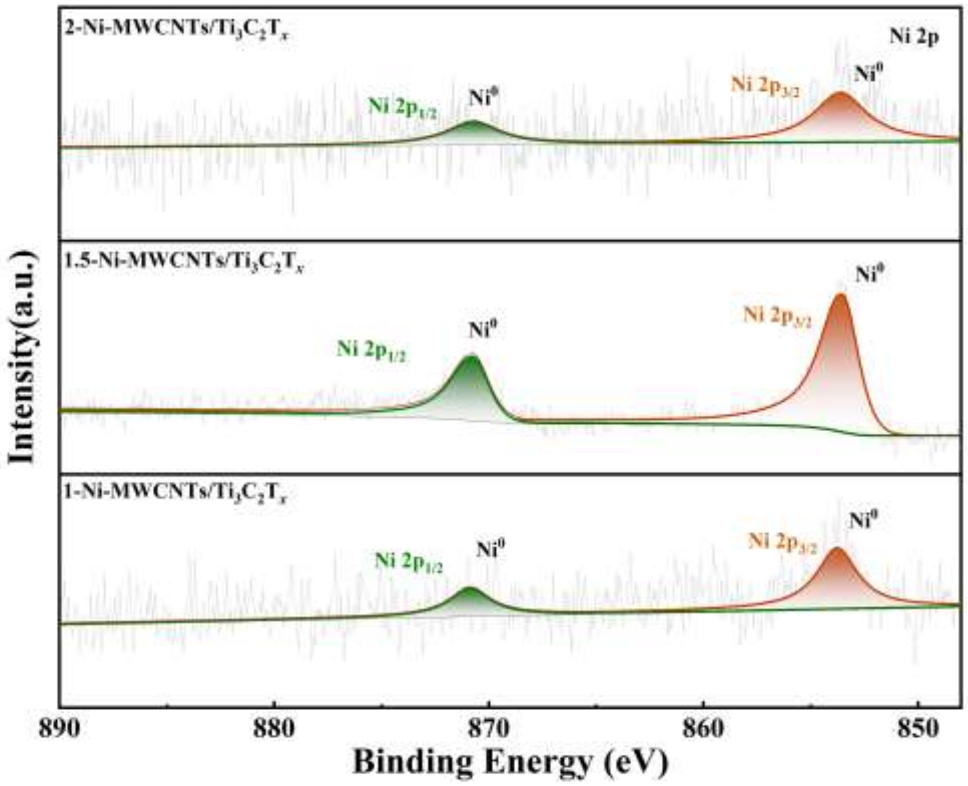

Fig. S11 XPS spectra of Ni 2p in Ni-MWCNTs/$Ti_3C_2T_x$.

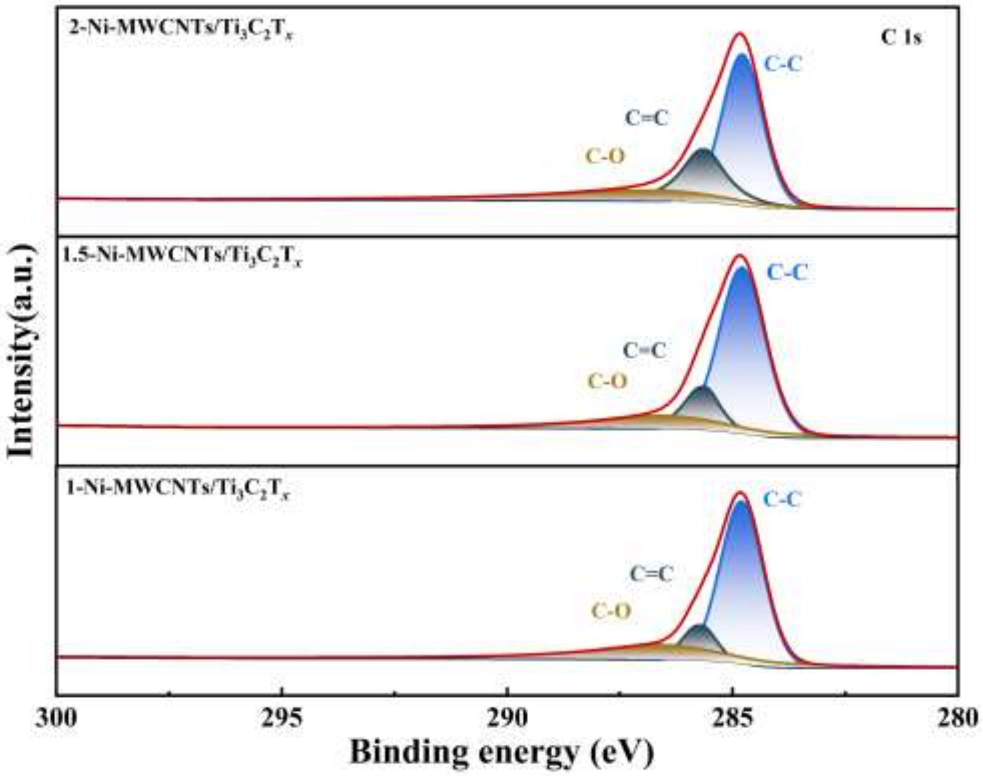

Fig. S12 XPS spectra of C 1s in Ni-MWCNTs/$Ti_3C_2T_x$.

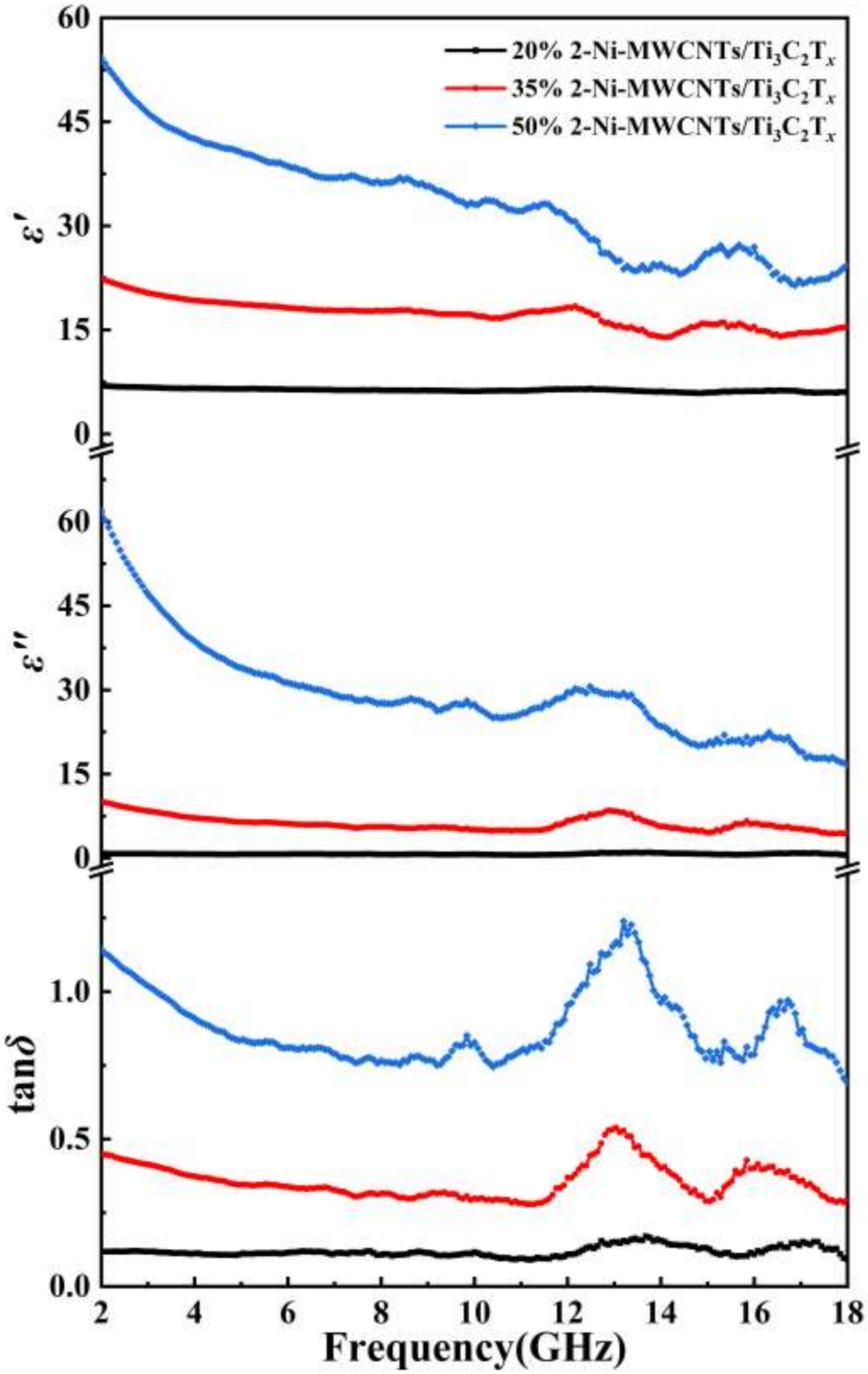

Fig. S13 Complex permittivity (real part (ε'), imaginary part (ε'') and dielectric loss (tan $\delta = \varepsilon''/\varepsilon'$) of 2-Ni-MWCNTs/$Ti_3C_2T_x$, in paraffin matrix with different loading.

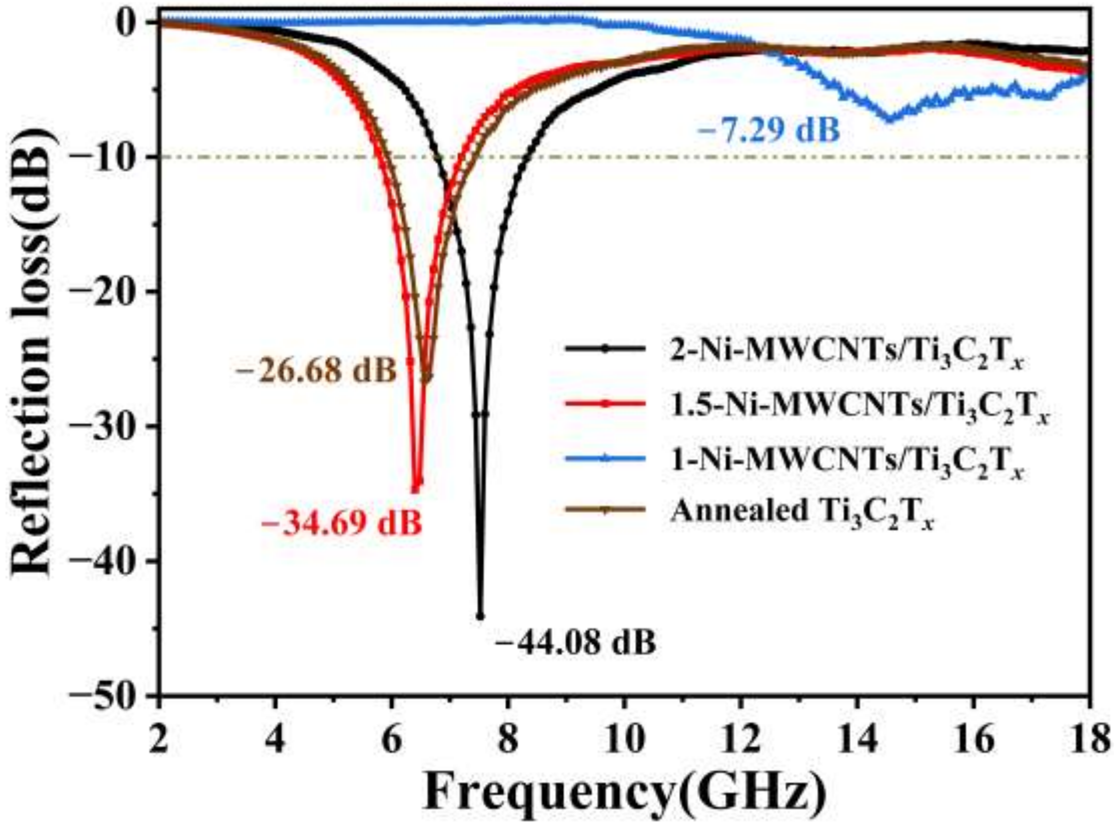

Fig. S14 Compared RL curves of 2-Ni-MWCNTs/$Ti_3C_2T_x$, 1.5-Ni-MWCNTs/$Ti_3C_2T_x$, 1-Ni-MWCNTs/$Ti_3C_2T_x$ and Annealed $Ti_3C_2T_x$.

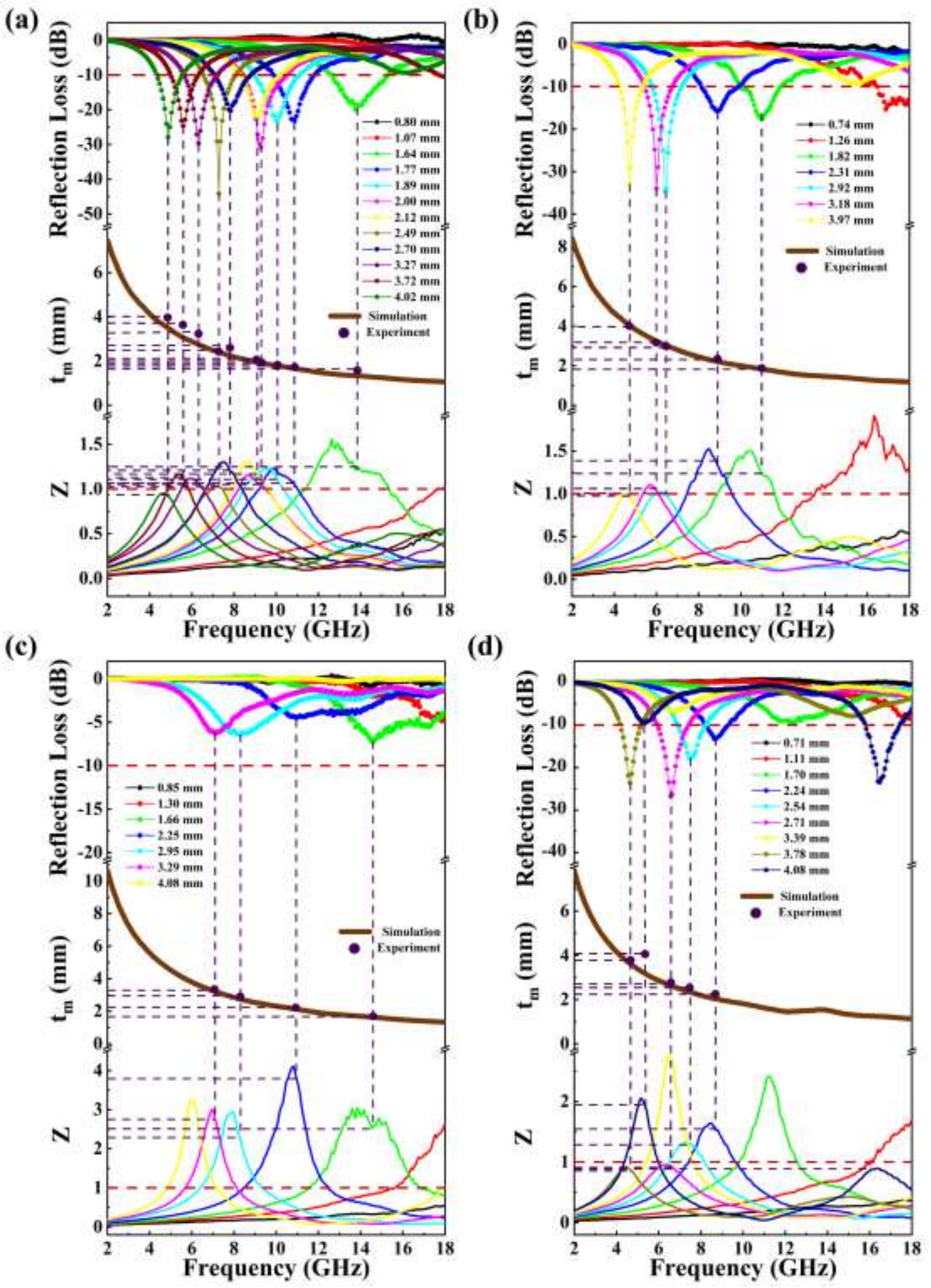

Fig. S15 RL-frequency curves, relationship between simulation thickness and peak frequency, and relationship between Z_{in}/Z_0 and electromagnetic wave frequency of **a** 2-Ni-MWCNTs/$Ti_3C_2T_x$, **b** 1.5-Ni-MWCNTs/$Ti_3C_2T_x$, **c** 1-Ni-MWCNTs/$Ti_3C_2T_x$, **d** Annealed $Ti_3C_2T_x$

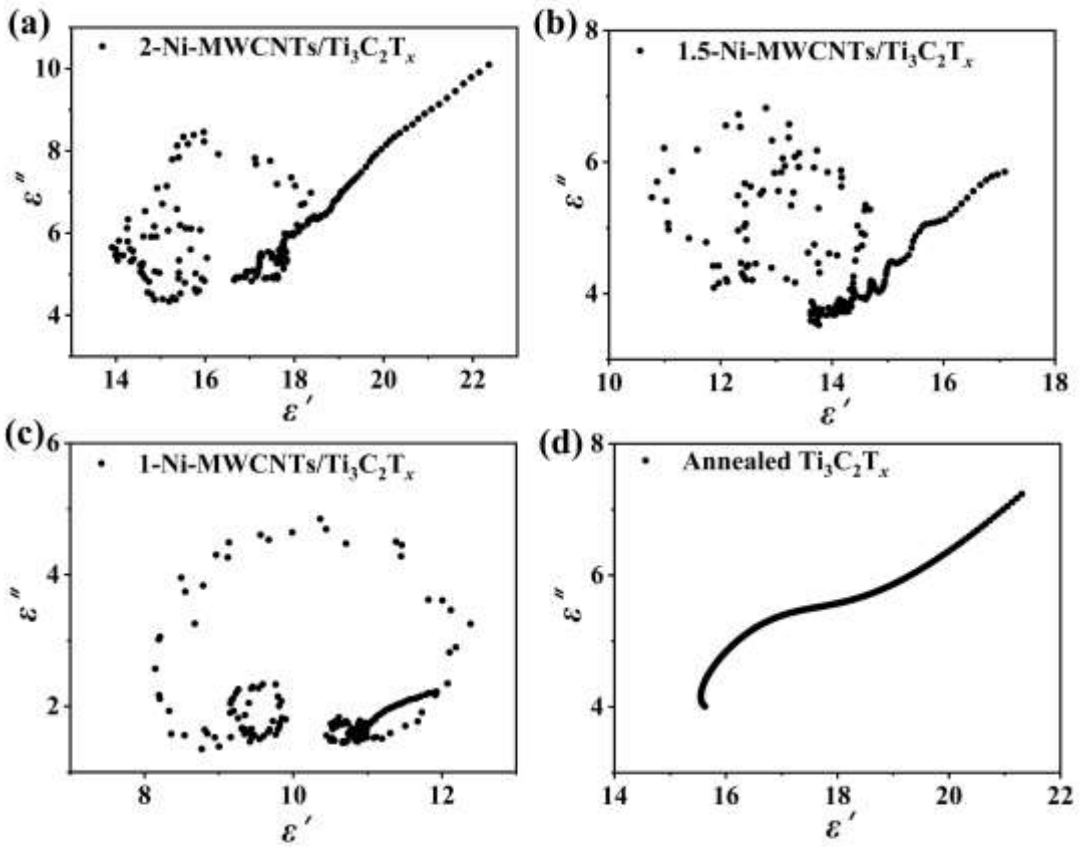

Fig S16 The Cole-Cole curves of **a** 2-Ni-MWCNTs/$Ti_3C_2T_x$, **b** 1.5-Ni-MWCNTs/$Ti_3C_2T_x$, **c** 1-Ni-MWCNTs/$Ti_3C_2T_x$, **d** Annealed $Ti_3C_2T_x$.
